# Differences in attitudes toward aging skin between skin of color and non-skin of color consumers: A cross-sectional survey study

**DOI:** 10.1016/j.jdin.2024.05.002

**Published:** 2024-05-17

**Authors:** Lynna J. Yang, Roopal V. Kundu

**Affiliations:** Department of Dermatology, Northwestern University Feinberg School of Medicine, Chicago, Illinois

**Keywords:** aging, anti-aging, colorism, cosmetic dermatology, esthetics, skin of color

*To the Editor:* Aging skin is a major concern, with nearly one-third of adults feeling dissatisfied with their skin.^1^ Skin of color (SOC) ages differently from non-SOC. Previous studies suggest that African American skin shows more noticeable sagging, and East Asian skin develops more hyperpigmentation.^2^ However, little research addresses how SOC consumers feel about aging skin.

A cross-sectional survey was administered online through REDCap. Eligible participants (English-speaking, ages 18 years or older) were recruited online through ResearchMatch. Statistical tests employed the chi-square test and Fisher’s exact test.

1434/1595 participants completed the survey (90% completion rate), including 37% (*n* = 532) SOC (self-reported Fitzpatrick skin types IV-VI) and 63% (*n* = 902) non-SOC (skin types I-III) (Table I).

**Table I:** Demographic information

Variable	*n* (%)
Age (y)	
18-30	216 (15.1)
31-50	408 (28.5)
51+	810 (56.5)
Gender	
Women	1125 (78.5)
Men	272 (19.0)
Nonbinary	37 (2.6)
Race/ethnicity	
Asian	83 (5.8)
Black or African American	166 (11.6)
Hispanic or Latinx	70 (4.9)
Native American or Indigenous	27 (1.9)
White	1014 (70.7)
Other	70 (4.9)
Marital status	
Single	420 (29.3)
Married or engaged	708 (49.4)
Divorced	210 (14.6)
Widowed	68 (4.7)
Other	28 (2.0)
Annual household income	
$0-24,999	145 (10.1)
$25,000-49,999	253 (17.6)
$50,000-74,999	294 (20.5)
$75,000-99,999	259 (18.1)
$100,000-199,999	363 (25.3)
$200,000+	120 (8.4)

Fifty-three percent of SOC and 32% of non-SOC participants report feeling happy about their skin (*P* < .0001) (Table II). Thirty-eight percent of SOC and 48% of non-SOC participants feel worse about their skin over time (*P* < .0001) (Table II).

**Table II:** SOC vs non-SOC perceptions toward aging skin

Question	Response	SOC *n* (%) Total *N* = 532 unless otherwise noted	Non-SOC *n* (%) Total *N* = 902 unless otherwise noted	*P* value
How do you feel about your skin?	Unhappy	123 (23)	324 (36)	<.0001
	Neutral	125 (23)	293 (32)	
	Happy	284 (53)	285 (32)	
How have your feelings about your skin changed over the last 5 years?	Less happy	201 (38)	434 (48)	<.0001
	Same	255 (48)	349 (43)	
	Happier	76 (14)	79 (9)	
How would you prefer to look compared to your current age?	Younger	372 (70)	606 (67)	.53
	Same as my age	157 (30)	289 (32)	
	Older	3 (1)	7 (1)	
How do you think you look for your age?	Younger	356 (67)	449 (50)	<.0001
	Same as my age	151 (28)	393 (44)	
	Older	25 (5)	60 (7)	
Age-related skin features rated as bothersome by those who have it.	Face: forehead wrinkles	183/325 (56)	441/668 (66)	<.01
	Face: smile lines	200/342 (59)	465/698 (67)	<.05
	Face: crow’s feet	189/309 (61)	451/683 (66)	.15
	Face: eye puffiness	277/324 (86)	556/639 (87)	.55
	Face: loose skin	230/275 (84)	499/582 (86)	.41
	Face: lip fullness	106/145 (73)	322/410 (79)	.21
	Face: uneven texture	268/316 (85)	527/636 (83)	.46
	Face: uneven tone	332/377 (88)	627/752 (83)	<.05
	Face: dryness	271/328 (83)	525/650 (81)	.54
	Hair: thinning	271/312 (87)	481/551 (87)	.92
	Hair: graying	239/416 (57)	410/721 (57)	.85
	Neck: loose skin	239/292 (82)	514/609 (84)	.34
	Neck: skin folds	209/241 (87)	410/492 (83)	.28
	Arms: loose skin	251/303 (83)	500/579 (86)	.16
	Arms: uneven tone	175/232 (75)	404/548 (74)	.65
	Arms: dryness	288/342 (84)	543/665 (82)	.34
	Arms: cellulite	157/180 (87)	312/364 (86)	.69
	Legs: loose skin	190/224 (85)	378/443 (85)	.91
	Legs: uneven tone	201/265 (76)	383/507 (76)	.99
	Legs: dryness	302/355 (85)	539/640 (84)	.78
	Legs: cellulite	304/338 (90)	592/665 (89)	.75
	Chest: uneven tone	179/215 (83)	354/456 (78)	.10
	Stomach: loose skin	332/355 (94)	602/639 (94)	.68
Factors that influence skincare routine	Cultural/societal perceptions of beauty	201 (38)	354 (39)	.61
	Colorism	65 (12)	10 (1)	<.0001
	Environment	85 (16)	140 (16)	.82
	Local climate	168 (32)	324 (36)	.10
	Pregnancy/breastfeeding	22 (4)	29 (3)	.38
	Prevention of future sun damage	248 (47)	548 (61)	<.0001
	Prevention of skin cancer	232 (44)	527 (58)	<.0001
	Social stigmas against skin conditions	76 (14)	126 (14)	.88
	Social stigmas against skin treatments	27 (5)	28 (3)	.07
	Social pressures	212 (40)	401 (44)	.10
	Treatment of current skin diseases	62 (12)	112 (12)	.74
	Work pressures	103 (19)	140 (16)	.07
Sources of skincare information	Advertisement	138 (26)	178 (20)	<.01
	Cosmetic/plastic surgeon consultation	27 (5)	57 (6)	.35
	Dermatologist consultation	196 (37)	443 (49)	<.0001
	Esthetician consultation at medical spa (Medispa) or spa	81 (15)	103 (11)	<.05
	Friends or family	222 (42)	357 (40)	.44
	Google or web search	285 (54)	445 (49)	.13
	Nurse practitioner (NP) or Physician’s assistant (PA) consultation	73 (14)	143 (16)	.29
	Social media influencer	75 (14)	78 (9)	<.01

*SOC*, Skin of color.

Similar proportions of SOC (70%) and non-SOC (67%) report a preference to look younger than their age (*P* = .53) (Table II). However, more SOC participants feel they look younger than their age (67%) than non-SOC (50%) (*P* < .0001) (Table II).

The top age-related skin concerns for both groups are loose stomach skin (94% for both) and leg cellulite (90% SOC; 89% non-SOC) (Table II). Uneven facial tone is a greater concern for SOC (88%) than non-SOC (83%) (*P* < .05) (Table II). Smile lines and forehead wrinkles are a greater concern for non-SOC (67% and 66%, respectively) than SOC (59% and 56%, respectively) (*P* < .05 and *P* < .01, respectively) (Table II).

Preventing future sun damage (47% SOC; 61% non-SOC) and skin cancer (44% SOC; 58% non-SOC) are the top skincare concerns for both groups but are greater concerns for non-SOC than SOC (*P* < .0001 for both) (Table II). Colorism impacted the skin care of 12% of SOC and 1% of non-SOC participants (*P* < .0001) (Table II).

The top sources of skincare information among all participants are internet search (54% SOC; 49% non-SOC) and family or friends (42% SOC; 40% non-SOC) (Table II). SOC participants (37%) are less likely to seek dermatologist consultation compared to non-SOC (49%) (*P* < .0001) (Table II). Instead, SOC participants are more likely to get their skincare information from advertisements (26% SOC; 20% non-SOC; *P* < .01), estheticians (15% SOC; 11% non-SOC; *P* < .05), or social media influencers (14% SOC; 9% non-SOC; *P* < .01) (Table II).

Limitations include the cross-sectional design and convenience sampling through ResearchMatch, which may limit generalizability.

While aging skin concerns all consumers, there are significant differences in the perceptions and preferences toward aging skin between SOC and non-SOC participants, emphasizing the need for tailored treatment approaches. The impact of colorism on SOC participants’ skin care reflects societal biases and highlights the importance of inclusivity and challenging discriminatory beauty standards in skincare education and marketing. Finally, the lower dermatologist consultation rate among SOC participants underscores the need for more accessible dermatological care and accurate skincare information for diverse skin types.

## Conflicts of interest

None disclosed.
